# Oral health status of children and young adults with maple syrup urine disease in Turkey

**DOI:** 10.1186/s12903-020-01376-0

**Published:** 2021-01-06

**Authors:** Elif Ballikaya, Yılmaz Yildiz, Nagihan Koç, Ayşegül Tokatli, Meryem Uzamis Tekcicek, Hatice Serap Sivri

**Affiliations:** 1 Department of Pediatric Dentistry, Faculty of Dentistry, Hacettepe University, 06230 Ankara, Turkey; 2 Section of Pediatric Metabolism, Department of Pediatrics, Faculty of Medicine, Hacettepe University, Ankara, Turkey; 3 Department of Dentomaxillofacial Radiology, Faculty of Dentistry, Hacettepe University, Ankara, Turkey

**Keywords:** Maple syrup disease, Dental caries, Gingival inflammation, Panoramic radiography, Dental anomaly, Diet, Turkey

## Abstract

**Background:**

Maple syrup urine disease (MSUD) is an inherited disorder clinically characterized by ketoacidosis, seizures, coma, psychomotor delay, and intellectual disability. The treatment requires a life-long protein-restricted diet, rich in carbohydrates and fats, supplemented with a medical amino acid formula. Diet, oral health and general health influence each other in a vicious cycle. The aim of this study was to investigate the oral health status of children and young adults with MSUD in Turkey.

**Methods:**

A descriptive study was conducted on patients with MSUD who applied for routine follow-up to the pediatric metabolic diseases clinic at Hacettepe University, Children's Hospital in Ankara, Turkey in a 12-month period. Patients with any other concomitant genetic diseases and acute infection were excluded. A total of twenty-five patients were enrolled and underwent oral examination including DMFT/S, dmft/s (decayed/missing/filled teeth/surfaces for deciduous and primary teeth, respectively), plaque and gingival indices. Panoramic radiographs were obtained in 12 cooperative patients.

**Results:**

Mean age was 9.88 ± 5.68 s.d years. More than half of the parents had only primary school level education, and low income. Fourteen patients consumed medical formula during or right before sleep. Fourteen patients reported caries-associated pain. Gingival inflammation was present in all 15 patients who cooperated for evaluation. Seven out of twelve patients had at least one dental anomaly or alterations in mandibular morphology. Five patients had previously been treated for caries under general anesthesia. To our knowledge, this is the first study to document oral clinical and radiologic findings in patients with MSUD.

**Conclusions:**

Impaired oral health was observed in this rare disease population. Regular dental referral by physicians, preventive measures and dental treatments should be included in multidisciplinary management of maple syrup urine disease to promote oral health.

## Background

Maple syrup urine disease (MSUD, OMIM #248600) is an autosomal recessive disorder resulting from the deficiency of branched-chain α-ketoacid dehydrogenase, which is involved in the degradation of branched-chain amino acids (BCAAs; leucine, isoleucine, and valine) and their branched chain α-ketoacid (BCKA) derivatives. Accumulation of leucine and α-ketoisocaproic acid causes metabolic encephalopathy, life-threatening brain edema, and progressive neurodegeneration. The disease is named after the classical maple syrup odor of body fluids associated with the concomitant increase of plasma isoleucine [1]. Early diagnosis and strict life-long dietary intervention prevent complications and may allow normal intellectual development. The intellectual outcome of patients with MSUD depends on duration of the elevated plasma leucine levels in the neonatal period and on the quality of long-term metabolic control [2]. Death most frequently occurs in metabolic crises which usually develop during the neonatal period or in association with acute infections. Therefore, timely evaluation and intensive treatment of minor illnesses is vital [3].

Treatment of MSUD mainly consists of a life-long strict semisynthetic diet to reduce the accumulation of toxic metabolites. Maintenance of normal physical development and nutritional status and preventing catabolism are other goals of disease management. Since toxicity arises from BCAAs, restriction of BCAA intake is the cornerstone of the therapy. Hence, a strict protein-restricted diet is prescribed, in which all animal products (meat, poultry, fish, eggs, dairy), legumes and nuts are forbidden, while grains and potatoes can be consumed only in limited amounts. Most fruits and other vegetables are acceptable for consumption. This protein-restricted diet must be supplemented with a BCAA-free amino acid supplement (special medical formula) to meet amino acid requirements. Such treatment aims to keep leucine, the primary toxic compound, below a threshold, while maintaining isoleucine and valine within a slightly higher range, which often requires additional supplementation of these two amino acids [4]. Additional fat and carbohydrate for energy requirements are provided by low-protein products and supplements like artificial medical formulas. Metabolic and nutritional status is evaluated at regular intervals [5]. Growth, development, and neuropsychological functioning are also monitored and supportive treatment is provided if necessary.

The prevalence of MSUD is variable among ethnic groups [6]. MSUD has been reported to be the most frequent organic acidemia in Turkey [7]. Although the exact incidence is not known (MSUD is not yet covered by the current newborn screening program), it is predicted to be as high as 1 in 50,000 newborns, considering the high rate of consanguineous marriages [8].

Phenylketonuria (PKU) is another inherited metabolic disease treated with a diet similar to MSUD: a low-protein diet supplemented with phenylalanine-free amino acid formulas consumed throughout the day and sometimes during the night [9]. Dental caries and gingival inflammation were evaluated in PKU patients in previous studies [10–12] because of the fact that these patients have a diet rich in carbohydrates and fats, which is considered to be risk factors for oral health. Patients with MSUD are also at high risk of dental caries, not only because they consume a similar, artificial diet, but also because they may have concomitant intellectual disability. Infections related to dental caries and poor oral health may aggravate the disease by leading to possible recurrence of metabolic crises. It is essential to describe the oral manifestations of patients with MSUD since there is only one report in the literature regarding the oral health status of this patient group with a high risk of developing complications [13]. This study aims to evaluate oral clinical and radiologic findings in MSUD patients, in terms of dental caries, gingival inflammation, oral health behaviors, and alterations in dental and mandibular morphology.

## Methods

This descriptive study was conducted to investigate the oral health status of children and young adults with maple syrup urine disease in Ankara, the capital city of Turkey, between March 2019 and February 2020. Within this period, 29 MSUD patients with no acute infection managed at Hacettepe University, Faculty of Medicine, Section of Pediatric Metabolism were consulted to Pediatric Dentistry Clinic for assessing the oral health status. MSUD patients with any other concomitant genetic diseases were excluded. Twenty-five MSUD patients between 2 and 28 years who were willing to participate were enrolled in the study. Based on history, physical examination, and hospital charts, an experienced metabolic physician completed a form regarding the data of medical history, level of intellectual disability or neurodevelopmental delay (hereby referred to as intellectual disability only), the amounts of protein supplied from natural foods and medical formulas.

Information about the level of intellectual disability was obtained from previously performed Bayley Scales of Infant Development-III [14], Stanford-Binet Intelligence Scales [15], Wechsler Intelligence Scale for Children-IV [16] or Wechsler Adult Intelligence Scale-IV [17], as appropriate for the patient’s age.

Data regarding oral health were obtained by an experienced pediatric dentist via a face-to-face questionnaire including sociodemographic features, diet, oral health complaints, oral health behaviors and via clinical evaluations. Clinical oral evaluations were performed using dental mirror and ball-ended explorer (WHO 973/80- Martin, Solingen, Germany) under dental unit lighting [18]. Diagnosis of dental caries was made according to codes and criteria determined by the World Health Organization (WHO), using dmft and dmfs indices for deciduous teeth, and DMFT and DMFS indices for permanent teeth, in which decayed (D), missing (M) and filled (F) teeth are evaluated and reported according to the number of teeth (DMFT and dmft) or surfaces (DMFS and dmfs) involved [18]. All teeth were examined under dental unit lighting in terms of enamel defects without drying before inspection. The modified developmental defects of enamel (mDDE) index scores were dichotomized regardless of the extent and type of enamel defect (whether demarcated opacity, diffuse opacity or hypoplasia) as 0 = no enamel defect, and 1 = with enamel defect (at least one surface) [19]. An enamel defect less than one mm was considered as sound [20, 21].

All four (mesial, distal, buccal and lingual) tooth surfaces were examined and evaluated according to the Löe gingival index (GI) [22] and Silness&Löe plaque index (PI) [23].Scores for each surface were added and the sum was divided by the total number of surfaces of examined teeth in order to obtain the individual GI or PI score. GI were classified as severe (2.1–3.0), moderate (1.1–2.0), mild (0.1–1.0) or no gingival inflammation [22]. Patients with moderate or severe gingival inflammation were also recorded as having gingivitis. Mean plaque index scores were registered between 0 and 3.

Digital panoramic images were obtained using either a Veraview IC5 device (Morita Corporation, Kyoto, Japan) or an Orthophos XG 5 device (Sirona Dental Company, Bensheim, Germany). Images with acceptable technical quality and providing adequate imaging quality for visualization of anatomical structures were included in the study. An oral and maxillofacial radiologist with seven years of experience evaluated the images for the presence of developmental dental anomalies such as aberrations in root or canal morphology (taurodontism, dilacerations, multiple canals, extra roots, fused roots, short roots and atypical shapes, etc.), agenesis, and Turner hypoplasia. Abnormalities in maxillofacial structures and alterations of bone morphology were also evaluated.

Statistical analysis was performed by using SPSS for Windows 21.0 (IBM Corp. Released 2012. Armonk, NY, USA). Number, percentage, mean, standard deviation, median, first and third quartiles and minimum and maximum values were calculated as descriptive statistics. Chi-square (χ^2^) test was used to assess the significance of the differences between categorical variables. The significance level was accepted as *p* < 0.05. The local Ethics Board for Non-Interventional Clinical Studies, and written parental informed consent was obtained.

## Results

### Demographic findings

A total of 25 patients were examined. Among these, 60% were male; the mean age was 9.88 ± 5.68 s.d years. Eighteen (72%) mothers and 13 (52%) fathers were primary school graduates and had not received further formal education. Seventeen families (68%) reported making minimum wage (under USD 360, low-income).

### Medical history and dietary habits

Only four (16%) patients had normal intelligence. Eleven (44%) patients had mild, four (16%) had moderate, and six (24%) had severe intellectual disability. Of the nine patients who had a comorbid condition, eight had epilepsy and one had asthma.

More than half of the patients had three main meals and additional 2–3 snacks a day. One of the patients was fed via nasogastric tube; all other patients were fed orally. Regarding nocturnal feeding, five patients consumed medical formulas before falling asleep at night, and nine patients consumed medical formula mid-sleep (Table 1). Nearly half of the patients (44%) had never received breast milk, eight (32%) had received breast milk for less than one month. The remaining patients had been breastfed up to 2, 6, 9, 12, 18, and 24 months.

**Table 1:** General characteristics of patients with MSUD

Characteristics	n	%
*Sex*		
Female	10	40
Male	15	60
*Age ranges (years)*		
2–6	7	28
7–12	10	40
≥ 13	8	32
X ± SD = 9.88 ± 5.68; 1.-3. Quartiles = (5.5–14); Min–Max = (2–26)		
*Education level of father*		
Illiterate	1	4
Primary	13	52
Secondary	3	12
High School	3	12
University	5	20
*Education level of mother*		
Primary	18	72
Secondary	4	16
High School	3	12
*Intellectual disability*		
No	4	16
Yes	21	84
Mild	11	44
Moderate	4	16
Severe	6	24
*Nutritional Habits*		
Main meal^a^		
2	5	20
3	17	68
4–5	2	8
Snack		
2–3	15	60
4–5	7	28
6–7	2	8
*Nocturnal feeding*		
No	11	44
Mid-sleep	9	36
Before asleep	5	20

^a^One of the patients feeds via a nasogastric tube eight times a day

### Dental history and oral hygiene habits

When the participants were questioned about the presence of oral health complaints, 14 (56%) reported having suffered from pain associated with dental caries, 11 (44%) reported halitosis, nine (36%) reported gingival bleeding, and two (8%) reported fractured teeth due to dental trauma. Eight (32%) patients had at least one bad oral habit such as nail biting, thumb sucking or bruxism. Seven patients aged 3–10 years still used baby-bottles for consuming formulas. Two patients did not own a toothbrush; 15 did not brush their teeth. Five patients brushed once, and five patients brushed twice a day.

### Clinical findings

**Fig. 1:**
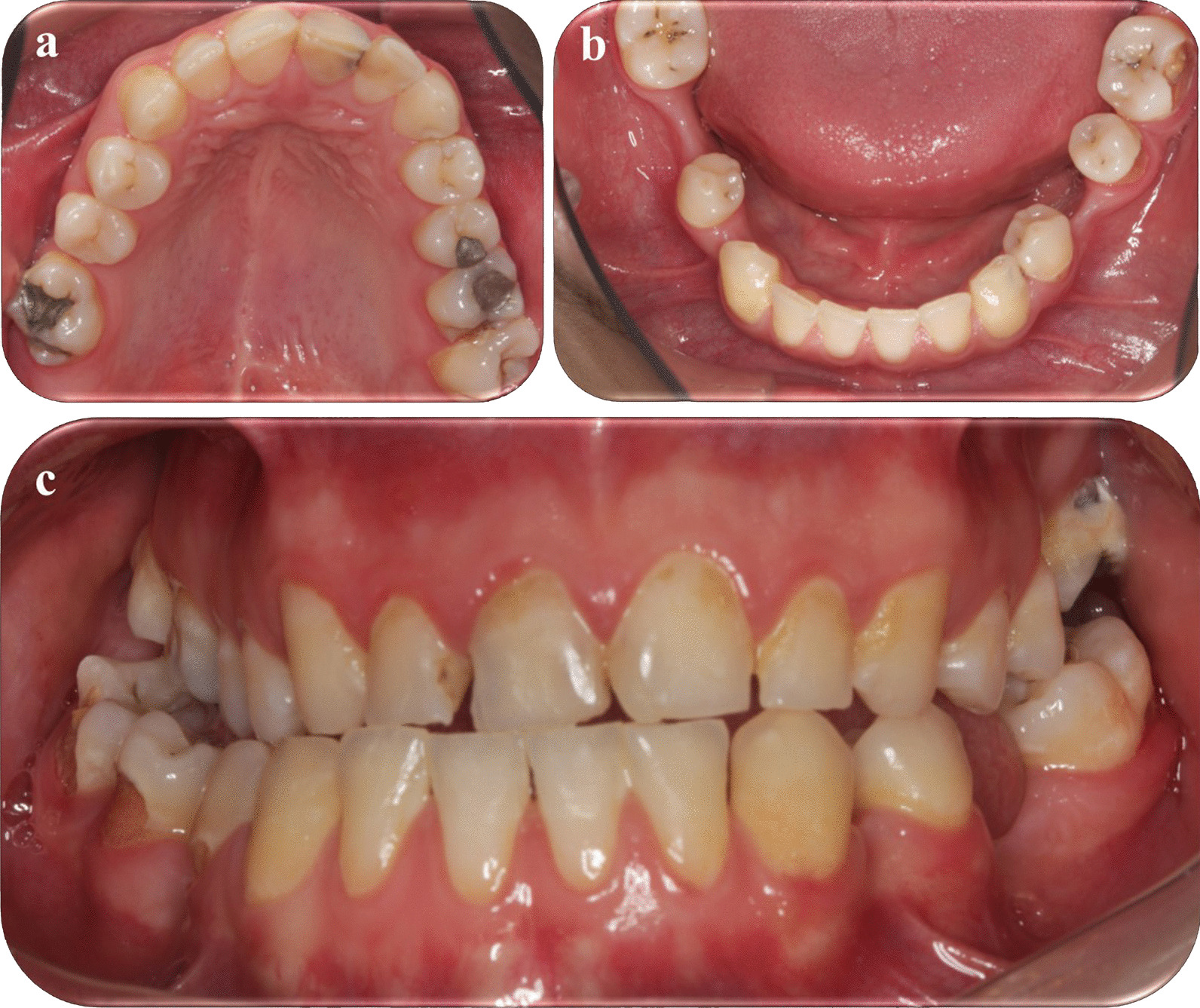
Oral features of a patient with MSUD (patient no.11): Multiple dental caries of permanent teeth, missing of permanent teeth due to previous dental caries (a, b:26,34,36,45,46), marginal gingivitis of maxilla and presence of fistula in left lateral incisor (**a**), malocclusion, narrow palatal arch and posterior crossbite (**a**–**c**)

In clinical examination (Table 2), dental caries was present in nearly three fourths (n = 18) of the patients. Considering dental caries by age, 14 out of 17 children (82.3%) under 12 years of age had dental caries. The mean dmft (sd) was 6.2 (4.37) and dmfs (sd) was 22.26 (18.67) (n = 15). The mean DMFT (sd) was 2.66 (18.67) and DMFS (sd) was 5.83 (8.2) (n = 18). Dental caries and gingival status could not be evaluated in one of the patients due to uncooperative behavior. In terms of mean plaque index scores, 31.6% (n = 6) of children had between 1.1 and 2, 68.4% (n = 13) had between 2.1 and 3 plaque index scores, that show all patients had visible dental plaque. Gingival inflammation levels of children were mild in 6.7% (n = 1), moderate in 86.7% (n = 13) and severe in 6.7% (n = 1). Among those with dental caries, five patients had history of dental procedures performed under general anesthesia. Figure 1 illustrates the clinical dental findings of a twenty-year-old patient with gingival, restorative, endodontic and orthodontic problems.

**Table 2:** Oral health-related findings of patients with MSUD

	n	%
*Dental caries (n = 24)*^a^		
Yes	18	75
No	6	25
*Enamel defects (n = 24)*^a^		
Yes	13	54.1
No	11	45.9
*Mean plaque index scores (n = 19)*^b^		
0–1	0	0.0
1.1–2	6	31.6
2.1–3	13	68.4
*Gingival inflammation levels (n = 15)*^c^		
Yes	15	100.0
Mild	1	6.7
Moderate	13	86.7
Severe	1	6.7

^a^One patient could not be evaluated due to uncooperative behavior

^b^Six patients could not be evaluated regarding plaque levels

^c^Ten patients could not be evaluated regarding gingival inflammation levels

### Radiographic findings

**Fig. 2:**
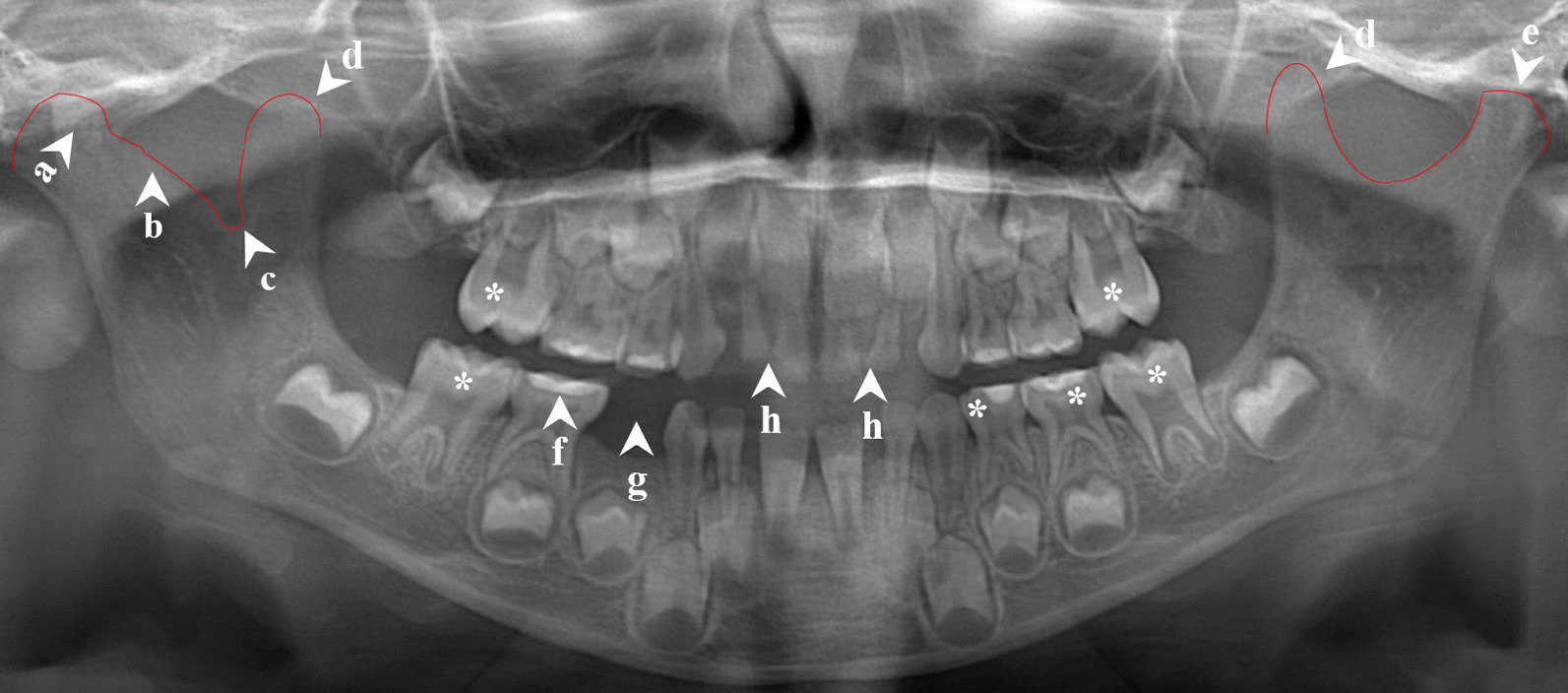
Radiological findings in a five-year-old patient with MSUD (patient no.10): Atypical morphology of the right mandibular condyle (**a**), condylar neck (**b**), and sigmoid notch (**c**). Bilateral coronoid hyperplasia (**d**) and flattening of the left mandibular condyle (**e**), taurodontism (asterisk), fillings in primary molars (**f**), missing primary molar (**g**) and early childhood caries (**h**)

Radiographic images with an adequate diagnostic quality (i.e., images without significant distortions, superimpositions, and artefacts) were obtained from 12 out of the 25 patients (Table 3). Of these, seven had alterations in the temporomandibular joint or coronoid process, which might lead to temporomandibular joint abnormalities. Seven had at least one dental anomaly, including aberrant mandibular premolar, Turner hypoplasia, or taurodontism. Periodontal bone loss was present in two patients. A summary of the radiological findings of a five-year-old patient is shown in Fig. 2.

**Table 3:** Radiological findings of patients with MSUD

Patient^a^	Age ranges	Aberrant mandibular premolar	Turner hypoplasia	Taurodontism	Alterations of mandibular morphology					
					Temporomandibular joint	Ramus	Coronoid process	Antegonial notch	Sigmoid notch	Mandibular canal
1	≥ 13	34	–	–	–	–	Hyperplasia (left)	–	–	Bifid (left)
2	7–12	34,35,45	–	–	Flattening of left mandibular condyle	–	–	–	–	Absence of cortication (left)
3	7–12	–	–	–	Flattening + sclerosis of left mandibular condyle	–	–	Prominent (left)	–	Bifid (left)
4^b^	7–12	–	25	–	Flattening of left mandibular condyle + erosion of right mandibular condyle	–	Hyperplasia (right)	Prominent (bilateral)	–	-
5	≥ 13	–	–	47, 36, 37, 26, 27, 17	–	–	–	–	–	–
6^c^	≥ 13	–	–	–	–	Short ramus height (left)	–	–	–	–
7	2–6	Not applicable	Not applicable	Not applicable	Flattening of mandibular condyles (bilateral)	–	–	Prominent (left)	–	–
8	7–12	–	24, 25	16, 26	–	Short ramus height (bilateral)	–	Prominent (left)	–	–
9	7–12	–	44, 34	–	–	–	–	Prominent (bilateral)	–	Absence of cortication (bilateral)
10	2–6	Not applicabl e	Not applicabl e	16, 26, 36, 46, 74, 75	Atypical right mandibular condyle and condylar neck + flattening of left mandibular condyle	–	Hyperplasia (bilateral)	–	Atypical morphology (right)	–
11^d^	≥ 13	–	–	–	–	–	–	Prominent (bilateral)	–	–
12	≥ 13	–	–	–	–	–	Hyperplasia (right)	–	–	Bifid (left)

^a^Thirteen patients could not cooperate for obtaining a panoramic image, therefore 12 patients could be evaluated

^b^The patient has also mandibular first primary molar with three roots

^c^Radicular cyst of left maxillary lateral incisor and impacted right maxillary second premolar are present

^d^Periapical lesion of left maxillary lateral incisor, generalized shortened roots and impacted maxillary wisdom teeth are also present

## Discussion

Diet plays an integral role in the maintenance of oral health. Dietary modifications due to medical problems such as PKU, in which therapeutic dietary practices are similar to MSUD, can have adverse effects on oral health. In the long run, the artificial composition of the consumed foods may have adverse consequences on health, including micronutrient or essential amino acid deficiencies, obesity, and reduced bone mineral density [24]. However, potential effects of the artificial diet on oral health of MSUD patients have not been extensively studied [13].

In the present study, a higher prevalence of dental caries was recorded in patients with MSUD under 12 years of age, compared to that obtained from a different study in healthy Turkish children (82.3% vs. 61%, respectively) [25]. As reported in the study of Topaloğlu et al. [26], a virtual palliative, preventive and restorative care is urgently required in juvenile oral healthcare in Turkey.

In 1989, Gazit et al. reported oral manifestations of a 14-year-old patient with MSUD [13]. They observed remarkable gingival inflammation, multiple rampant caries lesions mainly attributed to high carbohydrate MSUD diet, soft tissue and bone necrosis (alveolar osteomyelitis). The authors suggest that chronic destructive inflammatory process in the gingiva and local factors such as poor oral hygiene, heavy calculus formation, and severely decayed teeth could have possibly caused soft tissue and bone necrosis. Osteomyelitis is an uncommon but well-recognized complication of untreated odontogenic infection [27]. We did not encounter such a severe consequence of untreated caries in our patients.

Since there has been no other report on oral health indices of patients with MSUD, we could only compare the oral health results of MSUD with the data of patients with PKU. Studies evaluating the oral health status of children with PKU are few and have conflicting results [10–12]. Most studies in children with PKU have revealed a similar or lower prevalence of caries compared to healthy children. A low rate of caries despite a highly cariogenic diet might result from high phenylalanine (the accumulating amino acid in PKU), which has been considered to possibly act as a factor that limits the growth of plaque microorganisms [10, 11]. In a study performed at the same clinic, we have reported the prevalence of dental caries in 132 well-treated PKU patients as 67% [12]. Although dental caries in MSUD patients was higher than the PKU patients in that study; these differences might be attributed to the oral health behaviors, diet, and concomitant diseases such as epilepsy or intellectual disorders. It was reported in a previous study that intellectual performance of children with MSUD patients was significantly lower than that of a matched cohort of early treated PKU patients [28].

In a systematic review and meta-analysis regarding breastfeeding and dental caries; it was reported that breastfeeding up to 12 months of age is not associated with an increased risk of dental caries and in fact may provide some protection compared to formulas [29]. However, children breastfed for more than 12 months had an increased risk of dental caries due to prolonged breastfeeding, nocturnal feeding during sleep, cariogenic foods and drinks in the diet, or poor oral hygiene. In this study, most of our patients were breastfed for less than a year. Nocturnal feeding and no toothbrushing were present in more than half of the patients.

To the best of our knowledge, there has been no report of radiological findings in patients with MSUD in the literature. In the present study, panoramic radiographs revealed multiple abnormalities as shown in Table 3. Aberrant mandibular premolars, which were detected in two patients, is an uncommon anomaly of the root canal morphology indicating the presence of more than one canal in mandibular premolars. Aberrant root canal anatomy of mandibular premolars presents an endodontic challenge to successful treatment. There is an also increased probability of root fracture if such teeth are subjected to rotation during extraction [30]. It is important to be able to determine the morphology of these teeth radiologically and obtain a thorough knowledge of root canal anatomy and possible variations [31]. Seven out of 12 panoramic images had alterations in the temporomandibular joint or coronoid process. Although panoramic images could not be obtained in all of the patients due to intellectual disability or uncooperative behavior, given these images, possible temporomandibular symptoms such as restricted mouth opening, the clicking sound in temporomandibular joints should be considered in clinical examinations [32].

MSUD may be fatal if not treated, but once effective treatment is provided, long-term survival is good. However, intellectual impairment is frequently encountered in affected children [28]. It was reported that people with intellectual impairment are more likely to have poor oral hygiene and periodontal disease, and are possibly more likely to have caries than people without intellectual impairment [33, 34]. In this study, prevalence of intellectual disability, dental caries, and plaque index scores was found to be high which was in accordance with the current knowledge.

Children who have chronic conditions, intellectual disabilities, or other health problems are referred to as “children with special health needs”. Oral health is critical to good systemic health, especially in children with special health needs. Consequences of MSUD and its treatment have physical and psychological effects on children and their families, such as poor growth due to the restricted lifelong diet, motor disorders, and neurocognitive disorders [35, 36]. In a study regarding the health-related quality of life of the children and the parents of children affected with organic aciduria, urea cycle defects, or MSUD; the altered ‘physical’ and ‘social’ quality of life scores were comparable with patients with leukemia and their families [35]. In our study, fourteen patients had pain associated with dental caries. More than half of the parents did not receive an education after primary school level and reported making minimum wage. It has been reported that untreated dental caries with associated toothache have negative effects on weight gain, growth, and quality of life, as well as the cognitive development of young children [37]. The health-related quality of life of the parents and better care for their children could be managed by providing education and psychosocial support, which could also potentially improve dental health [38].

It was difficult to include a matched control group in this study because a lot of confounding factors were present, such as socioeconomic status of parents, dietary restrictions, intellectual disability and concomitant diseases. Although the sample size may seem small, which may be regarded as a limitation, and may not represent the oral health status of the whole MSUD population, it is also quite large for such a rare disease in a single center. Prospective cohort studies in larger samples, including children with MSUD and matched control groups may be necessary to clarify the further interaction between MSUD and oral health.

## Conclusions

This study suggests that patients with MSUD are at increased risk of impaired oral health. Most MSUD patients had dental caries, gingival inflammation, and enamel defects. Physicians have the opportunity and responsibility to refer MSUD patients to dentists to prevent oral complications of the disease and to promote proper oral health behaviors. The periodical dental check-ups and providing preventive approach may improve the oral health status of MSUD patients.

## Data Availability

The datasets generated and/or analysed during the current study are not publicly available, but are available from the corresponding author on reasonable request.

## Abbreviations

BCAA: Branched-chain amino acid
BCKA: Branched-chain α-ketoacid
DMFS: Decayed/missing/filled surfaces
DMFT: Decayed/missing/filled teeth
GI: Gingival index
mDDE: The modified developmental defects of enamel
MSUD: Maple syrup urine disease
PI: Plaque index
PKU: Phenylketonuria
WHO: World Health Organization
